# Molecular Diversity of the Antimicrobial Domain of Beta-Defensin 3 and Homologous Peptides

**DOI:** 10.1155/2009/983636

**Published:** 2009-11-02

**Authors:** Gerardo M. Nava, Magdalena Escorcia, M. Pilar Castañeda

**Affiliations:** ^1^Facultad de Medicina Veterinaria y Zootecnia, Universidad Nacional Autonoma de Mexico, Ciudad Universitaria, 04510 Mexico, D.F., Mexico; ^2^Department of Pathology & Immunology, School of Medicine, Washington University, Box 8118, 660 S. Euclid Avenue, St. Louis, MO 63110, USA

## Abstract

Human *β*-defensin 3 has received great interest for possible pharmaceutical applications. To characterize the biology of this antimicrobial peptide, the mouse *β*-defensin 14 has been selected as a prototypical model. This report provides definite evidence of true orthology between these defensins and reveals molecular diversity of a mammalian specific domain responsible for their antimicrobial activity. Specifically, this analysis demonstrates that eleven amino acid residues of the antimicrobial domain have been mutated by positive selection to confer protein niche specialization. These data support the notion that natural selection acts as evolutionary force driving the proliferation and diversification of defensins and introduce a novel strategy for the design of more effective antibiotics.

## 1. Introduction

A variety of antimicrobial peptides are produced by animals and plants as elemental components of their defense system to combat infectious microbes, including bacteria, fungi, viruses, and protozoa [1]. Among this antimicrobial protein repertoire, defensins are the most efficient and ancient components of host defense [2]. Recent analyses indicated that the cysteine-stabilized *α*-helical and *β*-sheet protein fold characteristic of the defensin family originated before the emergence of eukaryotes [3]. Even though defensins have an ancient origin, the majority of microbial pathogens have not developed highly effective mechanisms of resistance against these antimicrobial compounds [1, 2]. Antimicrobial resistance to defensins have been observed in some pathogens [4]; however, it has been proposed that the production of antimicrobial peptides and mechanisms of resistance have coevolved at similar rate, generating a niche specific transitory stage for host-pathogen balance, and this scenario has shaped the existing defensin repertoire [2, 5, 6]. To this end, the understanding of the evolutionary forces driving this molecular process should facilitate the design of more effective antibiotics.

Among all these antimicrobial peptides, human *β*-defensin 3 (DEFB103A) has received the most interest for possible pharmaceutical applications [7, 8]. This protein exhibits not only a broad-spectrum antibacterial effect [9] but also other important biological functions such as chemoattraction of immune cells [7] and initiation of remodeling processes in articular cartilage [10]. To identify and functionally characterize the role of DEFB103A peptide in host-microbe interactions, the putative homologous protein, the mouse *β*-defensin 14 (DEFB14) has been selected as a prototypical model [11–13]. Human DEFB103A and mouse DEFB14 carry the highest number of cationic charges (+11 and +12, resp.) of all *β*-defensins (BDEFs) [11]. The DEFB14 gene is constitutively expressed in epithelial cells of eye, tongue, trachea, esophagus, thymus, lung, liver, small intestine, spleen, testis, epididymis, and dendritic cells [11, 12].

The present report reveals the precise orthologous relationship between *β*-defensin 3 and homologous peptides and provides new insights into the protein niche specialization of these antimicrobial proteins. These findings provide important molecular bases for the design of more effective antibiotics.

## 2. Materials and Methods

### 2.1. Identification and Analysis of *β*-Defensin 3 Orthologous Groups

The widely used approach of reciprocal (bi-directional) best hits (RBH) [14, 15] was implemented for constructing related orthologous groups of DEFB103A and DEFB14 across multiple eukaryotic taxa. BLASTP analysis [16] was used to back-search amino acid sequences against well-annotated and -curated protein sequences from the RefSeq database (NCBI Reference Sequence Project). Protein sequences from this orthologous group were aligned using the M-Coffee software, a metamethod for assembling multiple sequence alignments [17]. Jalview software analysis [18] was used to estimate degree of conservation and consensus between all protein sequences. Then, the protein alignments were used for phylogenetic tree reconstruction using the maximum parsimony method [19] with the PAUP* 4.0 b10 program [20]. The statistical significance of branch order was estimated by the generation of 1000 replications of bootstrap resampling of the originally-aligned amino acid sequences.

### 2.2. Evolution of *β*-Defensin 3 in the Eutheria–Metatheria Clade

Because the identification of domains that occur within proteins can provide insights into their function [21, 22], the identification of conserved domains in protein sequences of the Eutheria–Metatheria BDEF3 (hereafter mammalian BDEF3) clade was analyzed. Pfam algorithm [23, 24] was used for the identification of conserved domains within the predicted mammalian BDEF3 orthologous group. Protein sequences of the mammalian BDEF3 orthologous group were aligned using the M-Coffee software and phylogenetic tree reconstruction by means of maximum parsimony was performed as described above.

### 2.3. Assessment of Protein Niche Specialization in the Mammalian BDEF3 Clade

To examine the likelihood of protein niche specialization in the mammalian BDEF3 clade, homologous proteins were aligned using the M-Coffee software and Jalview was used to identify the twenty-three residues, FLPKTLRKFFCRIRGGRCAVLNC, responsible for the antimicrobial activity [7, 8, 11]. This antimicrobial-domain was retrieved from each mature peptide region of proteins included in the mammalian BDEF3 orthologous group, and divergence of amino acid residues was analyzed by generating sequence logos [25]. To confirm the specificity of this antimicrobial-domain among the mammalian BDEF3 lineage, RBH was performed to identify other peptides containing this domain. BLASTP analysis was used to back-search amino acid sequences against the RefSeq database. In addition, site-specific synonymous and nonsynonymous substitution rate was estimated by maximum likelihood-based methods [26] for identification of residues subject to positive selection.

## 3. Results

### 3.1. Identification and Analysis of *β*-Defensin 3 Orthologous Group

**Figure 1:**
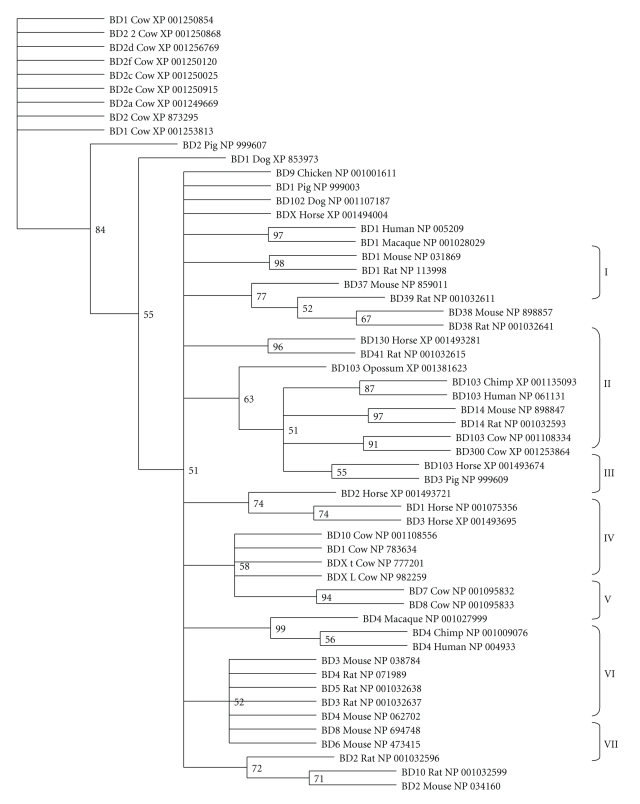
Phylogenetic tree of *β*-defensin orthologous groups. Maximum parsimony tree with bootstrap confidence levels based on protein sequences from the animal kingdom. Protein identifications correspond to *β*-defensin (BD) type, species, and accession number as reported by the RefSeq database. Seven major *β*-defensin clades are indentified: (I) rat-mouse BDEF38, (II) Eutheria–Metatheria BDEF3, (III) horse BDEF, (IV) cow BDEF, (V) primate BDEF4, (VI) rat-mouse BDEF4 and (VII) rat-mouse BDEF2. These results indicate that *β*-defensin has undergone niche specialization.

**Figure 2:**
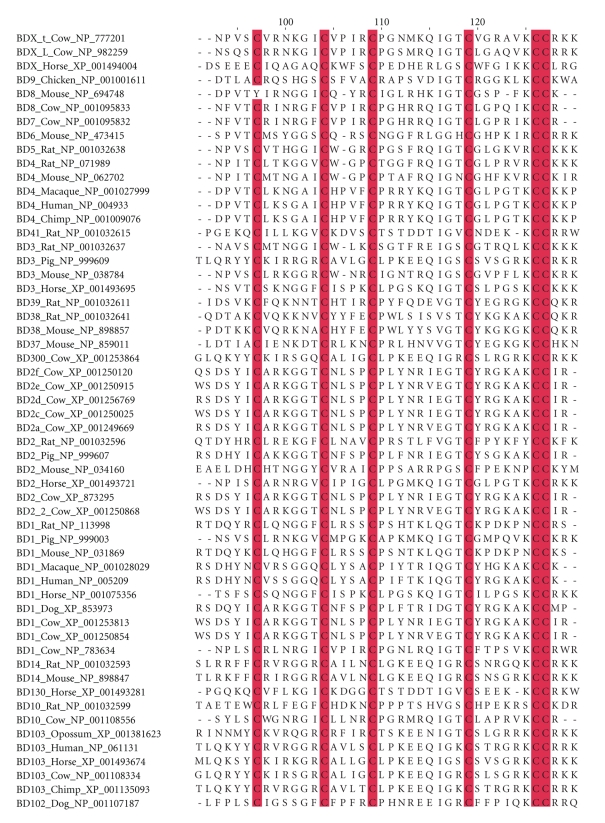
Conservation of the canonical six-cysteine motif in *β*-defensin orthologous groups. Multiple sequence alignment of complete *β*-defensin amino acid sequences was performed. Conserved cysteines in the mature peptide region are highlighted in red. Aligned residue position is indicated above the sequence. Only a fragment of the mature peptide region is shown.

Fifty-six BDEF protein sequences were obtained in the construction of DEFB103A and DEFB14 orthologous groups. This dataset enclosed protein sequences of BD1, -2, -3, and -4 across different taxa (Figure 1). Phylogenetic analysis using the maximum parsimony identified seven major BDEF clades: (I) rat-mouse BDEF38, (II) Eutheria–Metatheria BDEF3, (III) horse BDEF, (IV) cow BDEF, (V) primate BDEF4, (VI) rat-mouse BDEF4 and (VII) rat-mouse BDEF2 (Figure 1). Moreover, alignment and analysis of the BDEF proteins confirmed the conservation of the canonical six-cysteine motif in the BDEF orthologous group (Figure 2).

### 3.2. Evolution of *β*-Defensin 3 in the Eutheria–Metatheria Clade

**Figure 3:**
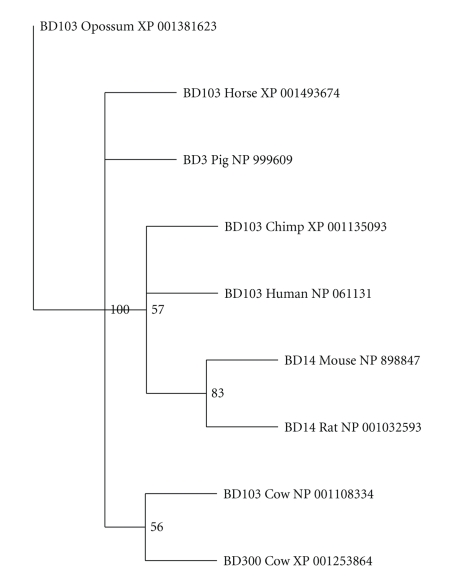
Phylogenetic tree of mammalian BDEF3 orthologous group. Maximum parsimony tree with bootstrap confidence levels based on protein sequences of mammals. Primate-, ungulate-, cow-, and rodent-BDEF3 clusters were identified. Protein charges are similar within sequences of each BDEF3 cluster (+12.2, +12 and +11, +10.7, +12, resp.). These results demonstrate that mammalian BDEF3 undergoes niche specialization during protein evolution.

To gain some insights into protein function of the mammalian BDEF3, the identification of conserved domains was performed with the Pfam algorithm [23, 24]. All proteins enclosed in the mammalian BDEF3 clade contained the structural components of the *β*-defensin domain (data not shown). Moreover, phylogenetic analysis revealed that protein similarity is greater among closely related species and identified primate-, ungulate-, cow-, and rodent-BDEF3 protein clusters (Figure 3). Interestingly, when cationic charges were estimated for each amino acid sequence, it was found that charges are similar within proteins of the primate-, ungulate-, cow-, and rodent-BDEF3 clusters (+12.2, +12 and +11, +10.7, +12; resp.).

### 3.3. Assessment of Protein Niche Specialization in the Mammalian BDEF3 Clade

**Figure 4:**
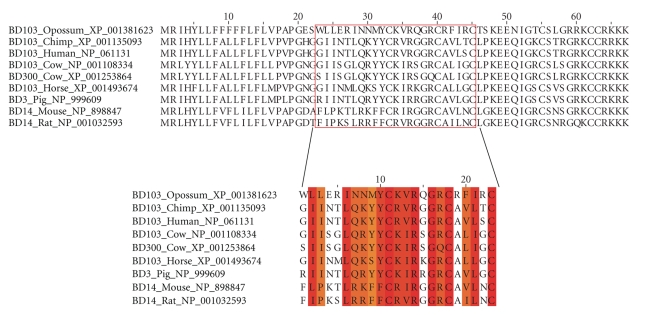
Mammalian specific antimicrobial domain. Proteins were aligned and the twenty-three residue antimicrobial-domain was localized (boxed sequence, top alignment) and retrieved (bottom alignment) from each mature peptide. These data reveals the divergence of amino acid residue in *β*-defensin 3 and homologous peptides.

**Figure 5:**
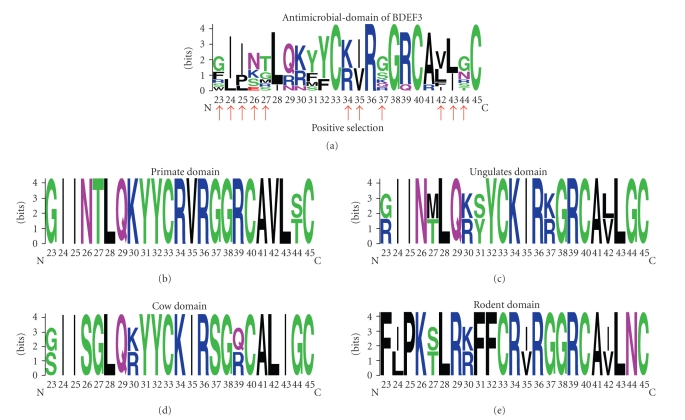
Sequence logos generated for each antimicrobial-domain showing the divergence of amino acid residue in *β*-defensin 3 and homologous peptides. Red arrows show amino acids subject to positive selection. Primate-, ungulate-, cow-, and rodent-BDEF3 clusters have different amino acid residue variations. This analysis demonstrates that eleven amino acid residues of the antimicrobial domain have been mutated by positive selection to confer BDEF3 niche specialization.

To gain some insights into the molecular basis for protein niche specialization of mammalian BDEF3, antimicrobial-domains were retrieved from each mature peptide region of proteins included in the mammalian BDEF3 orthologous group (Figure 4). Then, sequence logos were generated for each antimicrobial-domain identified (Figure 5). These analyses demonstrate that eleven out of twenty-three residues in the antimicrobial-domain are highly variable across the mammalian BDEF3 clade (Figures 4 and 5). Moreover, primate-, ungulate-, cow-, and rodent-BDEF3 clusters show different amino acid residue variation (Figure 5). To confirm the specificity of this antimicrobial-domain among the mammalian lineage, the amino acid residues contained in the antimicrobial domain were subject to RBH analysis with BLASTP algorithm against the RefSeq database. This analysis confirmed that this antimicrobial domain is a particular attribute of the mammalian BDEF3 clade. This unusual pattern is evidence of natural selection acting on the diversification of BDEFs and supports the idea of mammalian BDEF3 niche specialization. To confirm this notion, site-specific synonymous and nonsynonymous substitution rates were estimated by maximum likelihood-based methods [26] for the identification of residues subject to positive selection. This analysis demonstrates that eleven amino acid residues of the antimicrobial domain have been mutated by positive selection to confer BDEF3 niche specialization (Figure 5).

## 4. Discussion

In an effort to improve our understanding of the molecular basis regarding the mechanism of action of BDEF and facilitate the design new therapeutic agents, the present study examines the evolution of *β*-defensin 3 and its antimicrobial-domain. Herein, the phylogenetic analysis of the BDEF protein family across multiple species provides definite evidence of true orthology between human DEFB103A and mouse DEFB14. Moreover, it is revealed that positive selection has acted to diversify defensins and that mammalian BDEF3 undergoes niche specialization during protein evolution.

The nomenclature of BDEF proteins is complicated. Defensins have been annotated sequentially on discovery, and in some instances, orthologous proteins receive the same number [27]. However, there are cases such as human DEFB103A and mouse DEFB14, in which orthologous relationships are not so obvious. In the present study, the phylogenetic analysis of the BDEF protein family across multiple species identified seven major BDEF clades and established orthologous relationships among these proteins. For example, the Eutheria–Metatheria clade is comprised by human, chimp, and cow DEFB103A; mouse and rat DEFB14; and cow, horse, and pig BDEF3 proteins. Moreover, the phylogenetic analysis confirmed the conservation of the canonical cysteine motif (X_2-10_CX_5-6_G/AXCX_3-4_CX_9-13_CX_4-7_CCX_n_) in the defensin family [27]. Disulphide bridges generated between these conserved cysteines confer more resistance to bacterial proteolysis, even though the disulphide bridges are not essential for the antimicrobial activity [2, 7, 28, 29]. These results demonstrate that *β*-defensins likely evolved to encode similar functions among eukaryotic taxa but these protein sequences have undergone niche specialization.

In the present study, protein niche specialization of mammalian BDEF3 is supported by at least three molecular bases. First, phylogenetic analysis of proteins from mammalian BDEF3 clade identified primate-, ungulate-, cow-, and rodent- protein clusters within the mammalian BDEF3 clade. It was also found that proteins of each BDEF3 cluster have similar cationic charges. Second, analysis of amino acid residues of the antimicrobial-domain [7, 8, 11] revealed that this protein domain is a particular attribute of the mammalian BDEF3 clade. Third, it was found that eleven out of twenty-three residues in the antimicrobial-domain are highly variable across the mammalian BDEF3 clade and that these amino acids have been mutated by positive selection to confer BDEF3 niche specialization. A similar evolutionary scenario was determined for clusters of mammalian *α*-defensins [30] and other *β*-defensins [31, 32].

It can be hypothesized that the selection pressures on the evolution of defensins might have occurred to preserve an adaptive phenotype, increase functional divergence, and enhance microbe killing efficiency [27, 31]. In fact, it was demonstrated that amino acid substitutions at sites subject to positive selection increase the antimicrobial activity of BDEFs against bacterial pathogens [33]. Accordingly, it was suggested that positive selection at particular residues is involved in directing a new antimicrobial response against specific pathogens [33]. The value of these observations for biomedical research is also established by the elegant study by Antcheva et al. [34]. These authors demonstrated that the increase in antimicrobial activity of two homologous *β*-defensin 2 (human and macaque) is caused by amino acid residues subject to positive selection [34].

Together, these data are consistent with the notion that natural selection acts as evolutionary force driving the proliferation and diversification of defensins. Indeed, these results strongly support the hypothesis that BDEFs niche specialization is caused by host-pathogen coevolution [2, 31, 35]. Thus, this information has potential for the structure-guide design of novel antimicrobial peptides.

In summary, this report indicates that the production of antimicrobial peptides is a response to pathogen diversity and their coevolution generates niche specialization for maintaining a host-pathogen balance. These data support the notion that natural selection acts as evolutionary force driving the proliferation and diversification of defensins and introduce a novel strategy for the design of more effective antibiotics.
